# Concussion Characteristics in the National Hockey League Before and After the Introduction of Rule 48

**DOI:** 10.1001/jamanetworkopen.2023.44399

**Published:** 2023-11-22

**Authors:** Michael G. Hutchison, Alex P. Di Battista, Willem Meeuwisse, Jared M. Bruce, Ruben J. Echemendia, J. Scott Delaney, Paul Comper

**Affiliations:** 1 Faculty of Kinesiology and Physical Education, University of Toronto, Toronto, Ontario, Canada; 2 David L. MacIntosh Sport Medicine Clinic, Faculty of Kinesiology and Physical Education, University of Toronto, Toronto, Ontario, Canada; 3 National Hockey League, New York, New York; 4 Department of Biomedical and Health Informatics, University of Missouri–Kansas City School of Medicine; 5 Department of Neurology, University of Missouri–Kansas City School of Medicine; 6 Department of Psychiatry, University of Missouri–Kansas City School of Medicine; 7 Concussion Care Clinic, University Orthopedic Center, University of Missouri–Kansas City; 8 Department of Emergency Medicine, McGill University Health Centre, Montreal, Quebec, Canada; 9 Toronto Rehabilitation Institute, University Health Network, University of Toronto, Toronto, Ontario, Canada

## Abstract

**Importance:**

A rule prohibiting a direct hit to the lateral side of the head (ie, Rule 48–Illegal Check to the Head) was introduced in the National Hockey League (NHL) in the 2010-2011 season and refined in the 2011-2012 season to widen the application to include direct contact to all areas of the head.

**Objective:**

To compare the incidence and proportion of concussions that occurred following hits to the head before and after the implementation of Rule 48.

**Design, Setting, and Participants:**

This retrospective cohort study used a before and after design to evaluate concussions among NHL players in regular season games. Data from the 4 seasons preceding the implementation of Rule 48 (2006-2007 to 2009-2010) were compared with data from 5 seasons following its implementation (2014-2015 to 2018-2019). Data were analyzed from October 31, 2021, to November 30, 2022.

**Main Outcomes and Measures:**

The incidence and relative proportion of concussion characteristics and mechanisms before and after the introduction of Rule 48.

**Results:**

In the analysis of 688 concussions for NHL seasons spanning 2006 to 2010 (n = 231) and 2014 to 2019 (n = 457), there was an overall increase in the incidence of concussion between the 2 time frames, although the incidence of concussions due to hits to the lateral aspect of the head decreased from 1.6/100 games to 1.0/100 games (estimated difference, 0.6/100 games [90% credible interval (CrI), 0.30/100 games to 0.90/100 games). There was also a reduction of 18.8 (90% CrI, 13.0-23.7) percentage points in the proportion of concussions due to hits to the lateral aspect of the head from 80 of 231 concussions (34.6%) to 61 of 457 concussions (13.3%). The incidence of concussions following hits to the head from body checks using the shoulder, arm, and glove was similar before and following the implementation of Rule 48. However, the proportion was reduced 14.7 percentage points from 113 of 231 concussions (48.9%) before to 149 of 457 concussions (32.6%) after implementation of Rule 48 (90% CrI of the difference, 8.9-20.7 percentage points).

**Conclusions and Relevance:**

The findings of this cohort study of NHL players suggest that after the introduction of Rule 48, the incidence rate and proportion of concussions from contact to the lateral aspect of the head decreased, as did the proportion of concussions following direct hits to the head.

## Key Points

**Question:**

What are the incidence and proportion of concussions among National Hockey League (NHL) players following the implementation of Rule 48–Illegal Check to Head?

**Findings:**

In this cohort study of 688 concussions among NHL players, the incidence following hits to the head from body checks using the shoulder, arm, and glove were similar before and after the implementation of Rule 48. However, the incidence of concussions from hits to the lateral aspect of the head decreased by 0.6/100 games, and the proportion decreased by 18.8 percentage points.

**Meaning:**

These findings suggest that the number of concussions following direct hits to the lateral aspect of head was reduced following the implementation of Rule 48.

## Teaser

This cohort study compares the incidence and proportion of concussions among National Hockey League (NHL) players due to a hit to the head before (2006 to 2010) and after (2014 to 2019) the implementation of Rule 48 (Illegal Check to the Head).

## Introduction

It is well known that prevention strategies in certain circumstances can reduce the number and severity of concussions in sports.^1^ In recent years, several professional sports leagues have implemented rule changes with the goal of reducing head impacts and prohibiting certain high-risk behaviors. Some rules have been structural, such as the National Football League (NFL) moving the kicking team forward from the 30- to the 35-yard line for kickoffs to increase touchbacks, while other rules have focused on penalizing players or teams for specific behaviors. For example, Major League Baseball (MLB) implemented a rule to protect athletes from collisions at home plate; the NFL instituted a rule prohibiting a player from lowering his head to initiate helmet contact with an opponent; professional soccer implemented the assessment of a red card (ie, the ejection of a player) for intentional elbow-head contacts; and professional rugby lowered tackle height. Follow-up observational studies that have examined the effect of these rules^2,3,4,5,6^ found varied success at changing behavior or reducing the incidence of concussion and other injuries.

Identifying appropriate policy or rule changes to potentially reduce the risk of concussion appears to differ by sport. Ice hockey is a collision sport played within a confined space, often at high velocities and with rapid direction changes. At the professional level, video analysis has been adopted to improve the documentation and evaluation of the events and mechanisms involved in a concussion.^7,8,9^ Hutchison et al^9^ performed video analyses of concussions among National Hockey League (NHL) players over 4 seasons (2006 to 2010). Specifically, they found the most common mechanism of injury was direct contact to the head by the shoulder, elbow, or glove, occurring in 62% of concussions.^9^ Of these hits to the head, most (67.3%) were initiated by the shoulder.^9^ Notably, almost half of the hits to the head (46.8%) were classified as a contact to the lateral aspect of the head. Subsequently, Rule 48 was introduced in the 2010-2011 season: “A lateral or blindside hit to an opponent where the head is targeted and/or the principal point of contact is not permitted.”^10^ Following the initial season when Rule 48 was implemented, in the 2011-2012 season the specific language referring to a “lateral or blindside” hit was removed to broaden the scope of the rule, and was therefore modified to state that the head had to be targeted and the principal point of contact, rather than “and/or.” Language was also added regarding whether the player taking the hit had put himself in a vulnerable position “immediately prior to or simultaneously with the hit” or if head contact with an otherwise legal body check was “unavoidable.”^11^ However, until now there has not been an evaluation of the sequence of events leading to concussions in NHL players since the introduction of Rule 48.

This study aimed to compare the incidence and proportion of concussions among NHL players that occurred due to a hit to the head before (2006-2007 to 2009-2010 seasons) and after (2014-2015 to 2018-2019 seasons) the implementation of Rule 48. We hypothesized that after the implementation of Rule 48, there would be a decrease in both the incidence and proportion of concussions resulting from hits to the head.

## Methods

This retrospective cohort study was approved by the University of Toronto Health Sciences Research Ethics Board and an informed consent waiver was granted because deidentified data were used. The study followed the Strengthening the Reporting of Observational Studies in Epidemiology (STROBE) reporting guideline.

Team physicians from the NHL diagnosed concussions using the definition established by the Concussion in Sport Group.^12^ Description of events (eg, potential mechanism, period, time in game) were noted in medical records by medical personnel, and the corresponding video records were identified. To the extent that video of an event was available and in line with the event’s description leading to the concussion diagnosis, the events underwent video analysis. A total of 688 of 817 regular season concussions (84.2%) were identified with video documentation for analysis. The remaining events (129 of /817 [15.8%]) were not coded because of poor video quality, lack of adequate camera angles, or occurrence of the suspected concussive event outside of game play (ie, warm-up, practice, or training). Thus, these 688 events (231 in the 2006-2010 cohort and 457 in the 2014-2019 cohort) were used for the remaining quantitative analyses. Of the 129 events not coded, there was a greater percentage of events with video records in the 2006-2010 cohort (70 of 301 [23.3%]) compared with the 2014-2019 cohort (59 of 516 [11.4%]).

The corresponding digital recordings of concussion events included slow motion and multiple viewpoints. The coders (including M.G.H.) who were responsible for interpreting the digital recordings were trained using reliable methods previously used for examining concussions in the NHL^8^ and are aligned with prior projects examining situational characteristics and mechanism of injury.^7,9^ Variables for coding were similar between seasons, although some name changes occurred to variables over the data collection period. Table 1 provides a summary of relevant operational definitions. Coders viewed concussive events captured on video across NHL regular seasons throughout the study period. Coding for situational characteristics and mechanism of injury were performed by 2 independent coders. If the 2 independent coders disagreed about the situational characteristics or mechanism of injury and could not arrive at a consensus, a third coder was consulted to adjudicate and form a consensus. However, there were no events where the third coder was required.

**Table 1.** Operational Definitions

Term	Definition
Context	The context that precipitated the eventual injury. For example, did the injury involve contact with an opponent with the purpose of separating the opponent from the puck or move the position of the opponent (ie, body check)? Additional options include fall and/or trips, fight, incidental player contact, struck by puck, or other.
Contact from	Identifying the body part or object that first contacted the injured player. As an example, consider the situation whereby a player is struck in the torso by an opponent’s shoulder and then falls and strikes his head on the ice. In this situation the contact from is with the opponent’s shoulder.
Contact to	The body part that is hit during the contact from. In the aforementioned example, the body region of contact is the torso.
Hit from	Refers to the direction of impact by an individual or object relative to the position of the injured player. For example, if a player is body checked shoulder to shoulder by an opponent, then direction of impact most likely will be classified as side.
Hit to the head	Defined as body checks where contact from was by the shoulder, elbow, or gloves and contact to head, neck, and/or face.
Location	Perimeter refers to the structure (ie, boards and shielding) around the outside of the ice surface encompassing the side boards, behind the goal, side of goal, and corners, as well as the ice surface 10 feet toward the middle of the rink. Open ice refers to the interior portion of the ice not accounted for by the perimeter.

### Statistical Analysis

Data were analyzed from October 31, 2021, to Nvember 30, 2022. The primary objective of this study was to compare the incidence and proportion of concussions that occurred following hits to the head (defined as a body check where the shoulder, arm, or glove and/or fist contacts the opponent’s head) in the 4 seasons preceding the implementation of Rule 48 (2006-2010 cohort) with the 5 seasons following its implementation (2014-2019 cohort). The 2010-2011 to 2013-2014 seasons were not analyzed because Rule 48 was implemented in 2 phases, and modifications continued to be made after its initial adoption. A difference in proportions (expressed as percentage points [pp]) or incidence (expressed as events/100 games) was estimated from an intercept-only logistic regression; contrasts were created from the posterior intercept estimates corresponding to the 2006-2010 cohort and the 2014-2019 cohort. The inverse logit function was used to transform all parameters from the log-odds scale to the probability scale for results reporting. This modeling approach was also used to compare concussion mechanisms and characteristics. In addition to the estimate of the mean difference between groups, a 90% credible interval (CrI) was also reported. For model formulation, please see the eMethods in Supplement 1.

Gaussian priors were used to regularize posterior parameter estimates for each intercept (group). Mean (μ) and variance (σ) parameters of the form α ∼ Normal (μ, σ) varied by comparison to establish an a priori skepticism of differences between groups; prior simulations were used to tune prior parameters to expect a mean group difference of 0 pp with a SD of the difference of approximately 10 pp. The priors for the mean proportion of each variable were set to the proportions observed in the 2006-2010 cohort for both groups. For example, if a particular variable comprised 10% of all concussions in the 2006-2010 cohort, the prior was set at 10% for both groups in the logistic regression. This approach served to provide conservative estimates of the difference between groups. For prior sensitivity analyses supporting results for the main outcome—proportion and incidence of lateral hits to the head—please see the eMethods in Supplement 1.

All models were fit using the Hamiltonian Monte Carlo engine Stan modeling language, version 2.27,^13^ via R, version 4.1.0,^14^ and the RStudio integrated development environment, version 1.4.1717^15^ (R Project for Statistical Computing). All models were run across 4 chains at 2000 iterations per change. Convergence was measured using an updated Gelman-Rubin convergence test, with all chains converging to less than 1.01.^16^ The Rethinking book package, version 2.13,^17^ was used to interface between RStudio and RStan.^18^ Plots were created using the ggplot2,^19^ bayesplot, version 1.8.1,^20,21^ and tidybayes, version 3.0.4,^22^ R packages. Code used to analyze the data can be accessed via GitHub.^23^

## Results

During the 4 regular seasons from 2006-2007 to 2009-2010, 301 concussions were diagnosed in 4920 NHL regular-season games, with an incidence of 6.1/100 games. In the 5 regular seasons from 2014-2015 to 2018-2019, a total of 516 concussions were diagnosed in 6232 NHL regular-season games, resulting in an incidence of 8.3/100 games. This is a mean increase of 2.0 concussions per 100 games (90% CrI, 1.0/100 games to 3.0/100 games) between the 2014-2019 cohort and the 2006-2010 cohort.

### Game-Related Characteristics of NHL Concussions Before and After Implementation of Rule 48

In the 4 seasons before the implementation of Rule 48 (2006-2010), there were 231 regular season concussions with video data. They occurred equally across open ice and the perimeter (108 of 231 [46.8%] vs 123 of 231 [53.2%], respectively), primarily in the defensive zone (106 of 230 [46.1%]) and during the first period of play (117 of 231 [50.6%]). They most often occurred when the injured player was a forward (149 of 231 [64.5%]) and not in possession of the puck (puck possession, 46 of 231 [19.9%]); penalties were not called on most incidents (penalty called, 60 of 220 [27.3%]).

In the 5-year period following the implementation of Rule 48 (2014-2019), there were 457 concussions with video data. The game-related characteristics were similar to those observed before Rule 48, with a few notable exceptions. First, the perimeter became the predominant location of injury (290 of 455 [63.3%]); the proportion of concussions occurring in open ice decreased by an estimated 9.0 pp (90% CrI, 2.7-15.0 pp; 99.3% of the probability mass of the difference [PM] >0 pp). Second, concussions occurring in the first period of play were an estimated 13.4 pp lower following the implementation of Rule 48 (117 of 231 [50.6%] vs 158 of 440 [35.9%], respectively; 90% CrI, −7.5 to 19.7 pp; 100% PM >0 pp). Table 2 provides a complete overview of concussion characteristics as proportions for game-related concussions before and after the implementation of Rule 48. Incidences are found in Table 3, and a visualization of the difference in proportions and incidence rates before vs after Rule 48 for all game characteristics is depicted in the Figure.

**Table 2.** Concussion Characteristics as Proportions Before and After the Implementation of Rule 48

Characteristic	Raw value, No. (%)^a^		Modeled differences of posterior contrast estimates, mean (90% CrI), pp (n = 4000)
	2006-2010 (n = 231)	2014-2019 (n = 457)	
Location			
Open ice	108 (46.8)	167 (36.7)	−9.0 (−15.0 to −2.7)
Offensive	79 (34.3)	167 (36.5)	2.0 (−3.4 to 7.9)
Neutral	45 (19.6)	95 (20.8)	1.0 (−3.5 to 5.8)
Defensive	106 (46.1)	195 (42.7)	0.5 (−5.5 to 6.3)
Mechanism			
All body checks	173 (74.9)	307 (67.5)	−6.7 (−12.1 to −1.3)
Body checks to head	113 (48.9)	149 (32.7)	−14.7 (−20.7 to −8.9)
Shoulder	78 (33.8)	91 (20.0)	−12.1 (−17.5 to −6.8)
Glove	8 (3.5)	8 (1.8)	−1.0 (−2.5 to 0.5)
Arm	27 (11.7)	50 (11.0)	−0.7 (−4.4 to 2.9)
Lateral hit to head	80 (34.6)	61 (13.4)	−18.8 (−23.7 to −13.0)
Game situation			
First period	117 (50.6)	158 (35.9)	−13.4 (−19.7 to −7.5)
Puck possession	46 (19.9)	77 (16.9)	−3.0 (−7.9 to 1.4)
Penalty called	60 (27.3)	96 (21.0)	−5.5 (−10.7 to 0.1)
Position			
Forward	149 (64.5)	281 (61.5)	−2.7 (−8.8 to 3.4)
Defense	61 (31.4)	146 (31.9)	0.5 (−5.5 to 6.3)
Goalie	4 (2.1)	30 (6.6)	2.5 (1.0 to 4.1)

Abbreviations: CrI, credible interval; pp, percentage points.

^a^ The reported percentages were not always calculated from the cohort denominator due to the presence of missing data for certain variables.

**Table 3.** Concussion Characteristics Incidence Rate Before and After the Implementation of Rule 48

Characteristic	Raw values, concussions/100 games		Modeled differences of posterior contrast estimates, mean (90% CrI), pp (n = 4000)
	2006-2010	2014-2019	
Location			
Open ice	2.2	2.7	0.5 (0.0 to 1.0)
Offensive	1.6	2.7	1.0 (0.6 to 1.4)
Neutral	0.9	1.5	0.5 (0.2 to 0.8)
Defensive	2.2	3.1	1.0 (0.6 to 1.4)
Mechanism			
All body checks	3.5	4.9	1.4 (0.7 to 1.9)
Body checks to head	2.3	2.4	0.1 (−0.4 to 0.5)
Shoulder	1.6	1.5	−0.1 (−0.5 to 0.3)
Glove	0.2	0.1	0.0 (−0.1 to 0.1)
Arm	0.5	0.8	0.2 (0.0 to 0.4)
Lateral hit to head	1.6	1.0	−0.6 (−0.9 to −0.3)
Game situation			
First period	2.4	2.5	0.1 (−0.3 to 0.6)
Puck possession	0.9	1.2	0.3 (0.0 to 0.6)
Penalty called	1.2	1.5	0.3 (−0.1 to 0.6)
Position			
Forward	3.0	4.5	1.4 (0.8 to 2.0)
Defense	1.2	2.3	1.0 (0.6 to 1.4)
Goalie	0.1	0.5	0.2 (0.1 to 0.3)

Abbreviations: CrI, credible interval; pp, percentage point.

**Figure.**
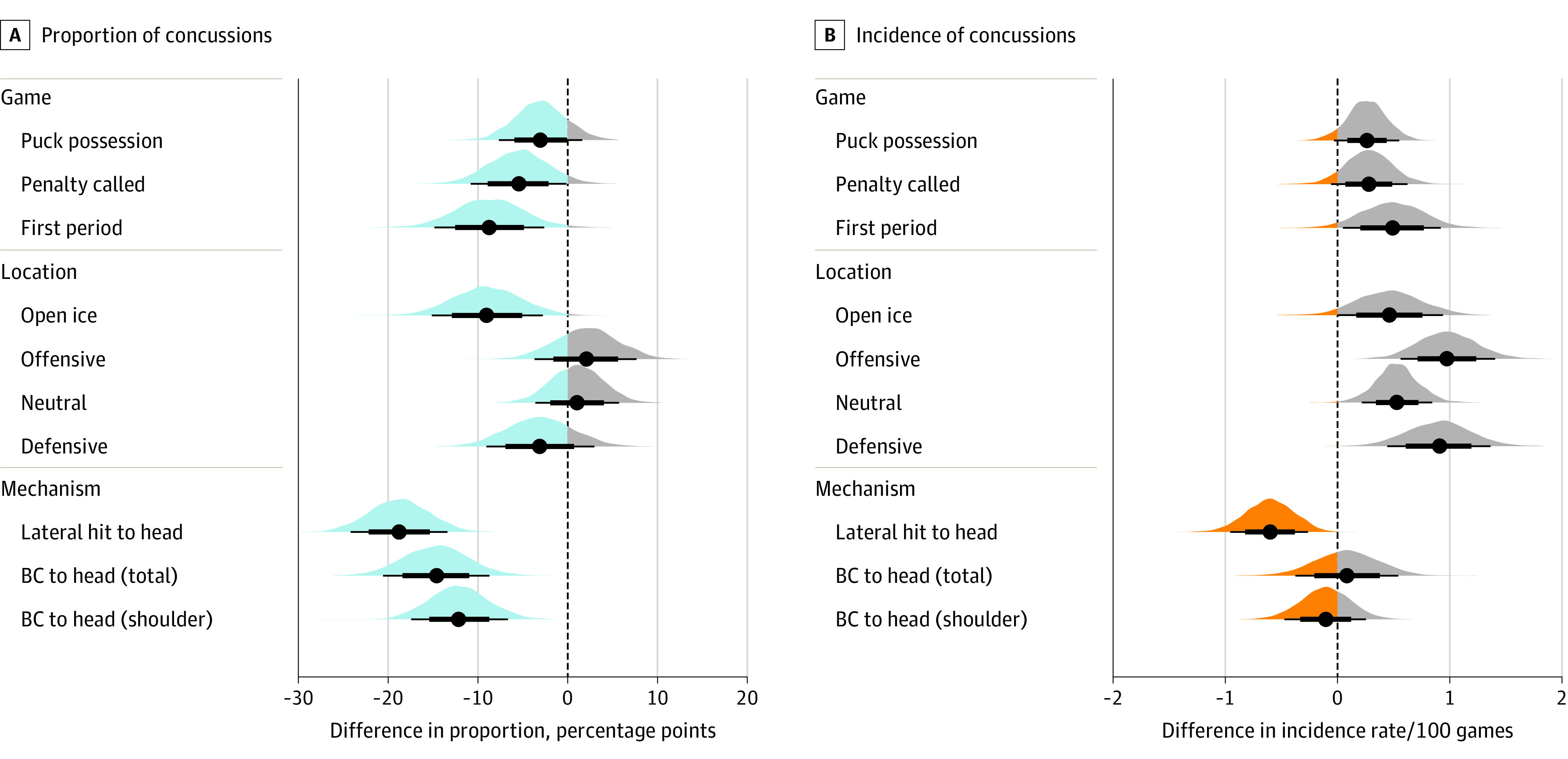
Difference in the Proportion and Incidence of Concussions Before and After Rule 48 The difference (contrast) in the proportion and incidence of concussions across characteristics relating to game situation, location, and mechanism in a cohort of National Hockey League players following the implementation of Rule 48 (2014-2019) compared with a cohort measured prior to its implementation (2006-2010). The vertical dotted line represents zero estimated difference between groups in both panels. A, Blue shading indicates the portion of the mass of the posterior distribution where there is a lower estimated proportion of concussions in the 2014-2019 cohort, while gray shading represents the portion of the mass of the posterior distribution where the proportion of concussions is higher in the 2014-2019 cohort. B, Orange shading indicates the portion of the mass of the posterior distribution where there is a lower estimated incidence of concussions in the 2014-2019 cohort, while gray shading represents the portion of the mass of the posterior distribution where the incidence of concussions is higher in the 2014-2019 cohort. Black dots represent the posterior mean estimate; the thicker black line, the 70% credible interval; and the thinner black line, the 90% credible interval. Plots are comprised of 4000 posterior sample draws. BC indicates body check.

### Comparison of Concussion Mechanisms in the NHL Before and After Implementation of Rule 48

Over the 4-season period prior to the implementation of Rule 48, concussions following hits to the head—defined as a body check where the shoulder, arm, or glove and/or fist makes contact with the opponent’s head—occurred at an incidence rate of 2.3/100 games; these 113 concussions in 4920 games accounted for 48.9% of 231 concussions. In the 5-season period following the implementation of Rule 48, the incidence rate was similar at 2.4/100 games (149 concussions in 6232 games). The difference was 0.1/100 games (90% CrI, −0.4/100 games to 0.5/100 games). However, the proportion of concussions from hits to the head was reduced by 14.7 pp (113 of 231 [48.9%] vs 149 of 455 [32.6%]; 90% CrI, 8.9-20.7 pp; 100% PM >0 pp).

In addition, concussions following hits to the lateral aspect of the head were reduced after the implementation of Rule 48 (1.6/100 games [61 concussions in 6232 games] vs 1.0/100 games [80 concussions in 4920 games]; difference, 0.6/100 games [90% CrI, 0.3/100 games to 1.0/100 games]; 99.9% PM>0). In addition, the proportion of concussions from hits to the lateral aspect of the head reduced by 18.9 pp (90% CrI, 13.0-23.7 pp; 100% PM >0 pp), from 80 (34.6%) of 231 to 61 (13.3%) of 455 concussions. Table 2 provides a complete overview of concussion characteristics as proportions for game-related concussions before and after the implementation of Rule 48. Incidences are found in Table 3, and the differences in proportion and incidence rates before vs after Rule 48 for all mechanisms are depicted in the Figure.

## Discussion

Concussion prevention remains at the forefront of many contact or collision sports, and rigorous evaluation of the potential sequences of events leading to a diagnosis of concussion is relevant to creating effective prevention strategies. The purpose of this cohort study was to compare the incidence and proportion of concussions that occurred among NHL players following a hit to the head in the seasons before (2006-2007 to 2009-2010) and after (2014-2015 to 2018-2019) the implementation of Rule 48. While the overall incidence of concussions increased during the 2 time frames analyzed, there were notable decreases in the incidence of concussions following hits to the lateral aspect of the head. Also, there was a meaningful reduction in the proportion of concussions following hits to the head overall, particularly direct hits to the lateral aspect of the head.

This study provides important insight regarding patterns of concussions and the potential influence of policies targeting health and safety. In the 2010-2011 season, Rule 48 was introduced to address specific behaviors preceding concussion.^10^ Subsequently, in the 2011-2012 season, the language referring to a “lateral or blindside” hit was removed to broaden the scope of the rule where the head is the targeted and principal point of contact.^11^ Over the most recent study period (2014-2019 cohort), implementation of Rule 48 was associated with a reduction in concussion diagnoses following targeted behaviors (eg, direct hits to the head).

Historically, there have been successful implementations of prevention strategies in the form of rule changes meant to decrease serious injuries in professional sports. Most notably, in the late 1970s, *spearing* was prohibited by the NFL to improve the head position of defensive players when tackling. The spearing rule was associated with a dramatic decrease in spinal cord injuries.^24^ More recently, the ability of these prevention initiatives to demonstrate a reduction in the incidence of concussions have been mixed.^2,3,4,5,6^ Although there was an overall increase in the incidence of concussions after the introduction of Rule 48, both the incidence and proportion of concussions from hits to the lateral aspect of the head decreased. While the findings of our study were limited to a professional sport, they underscore the association of policy implementation with behavioral change for the larger medical community. This opens avenues for contemplation among broader injury prevention strategies that harness policies and rules as tools.

Contrary to our hypothesis, the observed increase in concussion incidence was associated with several contributing factors. Importantly, the period following the introduction of Rule 48 coincided with enhanced efforts regarding concussion awareness and identification. This includes the addition of a spotter program whereby certified athletic trainers watch games centrally in an off-site location to identify visible signs of possible concussions. In-arena spotters also watch games live to identify visible signs, including those not captured by video. Assessment tools have improved and data interpretation approaches have been refined for use in the diagnosis of suspected concussion.^25,26^ An enhanced education campaign for all stakeholders on the health risks associated with concussion and the importance of concussion recognition (awareness) and early detection has also been implemented. These targeted initiatives may have resulted in greater awareness and reporting of concussion, thereby likely contributing to the increase in concussion incidence between the 2 periods.

The identification and prevention of concussion is multifaceted, with Rule 48 serving as one important component of a complex set of variables. Regardless, in recent years, it appears that most concussions occur around the perimeter of the ice rink (eg, side boards, corners). Thus, careful examination of the sequence of events and injury characteristics in the perimeter is warranted, particularly regarding the interaction of the playing environment (boards and shielding) and player-to-player contact. Player-to-player body contact remains the primary mechanism preceding concussion diagnosis. Given the inherent nature of collisions—both intentional body checks and incidental collisions—future research should involve a closer examination of the behaviors and mechanisms of concussions following player-to-player contact around the perimeter of the arena (eg, types of surfaces and environment, specific location of head contact). Also, research examining modifiable risk factors that may help to inform future primary prevention strategies may be warranted.

### Strengths and Limitations

A strength of this study is the large sample size and design: a longitudinal cohort of 688 physician-diagnosed concussions with video footage of the event enabled a careful examination of the sequence of events and mechanisms leading to concussion among NHL players. However, we were limited to events where video content of the event was available. During the study period, we identified and analyzed 84.2% of all reported concussions. While this proportion is similar to those of prior video analysis studies in the NHL,^7,9^ there were proportionally more events of poor quality or missing video content in the earlier study period (2006-2010); this is likely due to improved quality and/or additional cameras monitoring games in recent seasons. We acknowledge the potential bias, as these events may differ in their characteristics from the concussions with video. Additionally, our study focused exclusively on video events during the regular season and might not offer a complete picture of the new rule’s implications. By not considering events from preseasons and playoffs, we may not have captured the rule’s broader effects across various game scenarios. Last, a limitation of this study relates to the evolution of concussion awareness and evaluation protocols over the study period. This limitation is aligned with the observed increase in the incidence of concussions between the 2006-2010 and 2014-2019 cohorts, underscoring the evolving nature of concussion evaluation standards and awareness.

## Conclusions

The findings of this cohort study suggest that although the overall incidence of concussions among NHL players increased following the implementation of Rule 48, the incidence and proportion of concussions following direct hits to the lateral aspect of the head was reduced. Collectively, these findings further indicate that the introduction of Rule 48 has coincided with a change in player behavior leading to concussions. Continued efforts to identify meaningful primary prevention are still required, and future efforts should focus on both exploring and evaluating concussion prevention strategies targeting modifiable risk factors.

## Notes

Supplement 1. **eMethods.** Model Formulation and Statistical Analysis

Supplement 2.
Data Sharing Statement
