# Supplementary material for: Concussion Characteristics in the National Hockey League Before and After the Introduction of Rule 48
Source: JAMA Netw Open. 2023 Nov 22;6(11):e2344399. doi: 10.1001/jamanetworkopen.2023.44399 (PMC10665971; doi:10.1001/jamanetworkopen.2023.44399)
Supplement: Supplement 1. — eMethods. Model Formulation and Statistical Analysis [file jamanetwopen-e2344399-s001.pdf]

Supplementary Online Content

Hutchison MG, Di Battista AP, Meeuwisse W, et al. Concussion characteristics in the National Hockey League before and after the introduction of Rule 48. *JAMA Netw Open*. 2023;6(11):e2344399. doi:10.1001/jamanetworkopen.2023.44399

eMethods. Model Formulation and Statistical Analysis

This supplementary material has been provided by the authors to give readers additional information about their work.

© 2023 Hutchison MG et al. *JAMA Network Open*.

eMethods. Model Formulation and Statistical Analysis

$$y \sim \text{Binomial}(1, p)$$
$$\text{logit}(p_i) = \alpha_{\text{GROUP(pre/post)}}$$
$$\alpha \sim \text{Normal}(\mu, \sigma,)$$
$$\mu = \text{to be determined}$$
$$\sigma = \text{to be determined}$$

Model Formulation. The above logistic model formula was used to estimate the difference in incidence and proportion of concussions that occurred following hits to the head before and after the implementation of Rule 48, as well as other concussion mechanisms and characteristics. A varying-intercept model was used to estimate the outcome variable (y), which was the log odds of either the incidence or proportion of concussions occurring, depending on the data being modelled. The predictor variables (hits to the head, concussion mechanisms and characteristics) were then estimated according to group membership, with α_1 = pre-Rule 48 (2006 – 2010), and α_2 = post-Rule 48 (2014 – 2019). Posterior estimates were converted to probabilities by calculating the inverse of the log odds. Then, contrasts were created ($\alpha_2 - \alpha_1$) to estimate the difference in either the incidence or proportion between the two time periods. Priors varied according to the predictor variable being modelled.

Sensitivity Analysis for lateral hits to the head

Proportions

The prior used in the manuscript was a normal (mean = -0.65, sd = 0.3) on the log odds scale, which converts to ~35% with a 1SD range of approximately 27% to 41% on the probability scale. The posterior estimate for the difference between 2006-2010 and 2014-2019 was a mean decrease of ~19 percentage points from ~35% to 16%. A sensitivity analysis was performed spanning a range of *a priori* estimated proportions of lateral hits to the head from 20% to 50% (35% +/- 15%). Using a 50% prior (normal[0,0.3] on the log odds scale), the estimated difference was 20 percentage points; 37% to 17% before and following rule implementation. Using a 20% prior (normal[-1.5,0.3] on the log odds scale), the estimated difference was 17 percentage points; 31% to 14% before and following rule implementation. Hence, a span of possible prior proportions of lateral hits to the head pre-Rule 48 ranging from 20% to 50% (the raw data from 2006 – 2010 was ~35%) only corresponded to a posterior contrast estimate ranging three percentage points (17 – 20 percentage points).

Incidence

The prior used in the manuscript was a normal (-0.4,0.4) on the log odds scale, which converts to ~1.6/100 games with a 1SD range of approximately 1.2 to 2.7 / 100 games on the probability scale. The posterior estimate for the difference between 2006-2010 and 2014-2019 was a mean decrease of 0.6/100 from 0.3 to 0.9 / 100 games. A sensitivity analysis was performed spanning a range of *a priori* estimated incidence of lateral hits to the head from 0.5 to 2 / 100 games. Using a 0.5/100 games prior (normal[-5.2,0.4] on the log odds scale), the estimated mean

© 2023 Hutchison MG et al. *JAMA Network Open*.

difference was 0.6/ 100 games; 1.5 to 0.9 / 100 games before and following rule 48 implementation. Using a 2/100 games prior (normal[-3.9,0.3] on the log odds scale), the estimated difference was also 0.6/100 games; 1.6 to 1 / 100 games before and following rule 48implementation. Hence, changing the prior incidence by over 25% in either direction yielded the same estimated difference reported in the main manuscript.

© 2023 Hutchison MG et al. *JAMA Network Open*.
