# Supplementary material for: Concussion Characteristics in the National Hockey League Before and After the Introduction of Rule 48
Source: JAMA Netw Open. 2023 Nov 22;6(11):e2344399. doi: 10.1001/jamanetworkopen.2023.44399 (PMC10665971; doi:10.1001/jamanetworkopen.2023.44399)
Supplement: Supplement 2. — Data Sharing Statement [file jamanetwopen-e2344399-s002.pdf]

Data Sharing Statement

Hutchison. Concussion Characteristics in the National Hockey League Before and After the Introduction of Rule 48. *JAMA Netw Open*. Published November 22, 2023.
doi:10.1001/jamanetworkopen.2023.44399

Data
Data available: No
